# Primary care Physicians’ perspective on the management of anxiety and depressive disorders: a cross-sectional survey in Emilia Romagna Region

**DOI:** 10.1186/1471-2296-14-75

**Published:** 2013-06-07

**Authors:** Federica Casini, Cecilia Sighinolfi, Paola Tedesco, Pier Venanzio Bandieri, Maria Bologna, Niccolò Colombini, Clara Curcetti, Michele Magnani, Mara Morini, Alberto Serio, Ilaria Tarricone, Domenico Berardi, Marco Menchetti

**Affiliations:** 1 Institute of Psychiatry, University of Bologna, Viale C. Pepoli 5, IT-40123 Bologna, Italy; 2 Mental Health Department, Local Health Unit Rimini, Rimini, Italy; 3 Mental Health Department, Local Health Unit Reggio Emilia, Reggio Emilia, Italy; 4 Mental Health Department, Local Health Unit Modena, Modena, Italy; 5 Service for Health District, Primary Care, Planning and Development of Health Services, Regional Health Care and Social Authority, Bologna, Emilia Romagna Region, Italy; 6 Primary Care Department, Local Health Unit Bologna, Bologna, Italy; 7 Primary Care Physician of the Local Health Unit Bologna, Bologna, Italy

**Keywords:** Anxiety, Depression, Primary care, Antidepressants, Psychotherapy

## Abstract

**Background:**

Evidences from literature suggest that Primary Care Physicians’ (PCPs) knowledge and attitude about psychological and pharmacological treatments of anxiety and depressive disorders could influence their clinical practice. The aim of the study is double: 1) to assess PCPs’ opinions about antidepressants (ADs) and psychotherapy for the management of anxiety and depressive disorders; 2) to evaluate the influence of PCPs’ gender, age, duration of clinical practice, and office location on their opinions and attitudes.

**Methods:**

This cross-sectional multicentre survey involved 816 PCPs working in four Local Health Units of the Emilia Romagna Region. Participating PCPs were asked to complete a questionnaire during educational meetings between October 2006 and December 2008.

**Results:**

The response rate was 65.1%. Eighty-five percent of PCPs agreed on the effectiveness of ADs for depressive disorder whereas lower agreement emerged for anxiety disorder and on psychotherapy for both anxiety and depression. Forty percent of PCPs reported to feel “very/extremely confident” in recognizing depression and 20.0% felt equally confident in treating it with pharmacotherapy. Considering anxiety disorder, these proportions increased. Female PCPs and those located in the rural/mountain areas reported to adopt more psycho-educational support compared to male and suburban colleagues.

**Conclusions:**

Our results suggest that an effort should be made to better disseminate recent evidences about the management of anxiety and depressive disorders in Primary Care. In particular, the importance of psychological interventions and the role of drugs for anxiety disorder should be addressed.

## Background

Anxiety and depressive disorders are highly prevalent in the general population [1] and associated with disability and low quality of life [2,3]. Patients suffering from these conditions are usually treated by Primary Care Physician (PCP) while referral to mental health specialist should be considered for severe cases. Patients prefer to receive care from a regular family doctor into a trusting relationship and to avoid stigma related to mental health services access [4]. However, despite several evidence-based treatments for anxiety and depression are available [5,6], their application in the Primary Care setting has been troublesome. Although recent studies showed an increased number of PCPs using antidepressants (ADs) [7], other investigations found that only a minority of Primary Care attenders with anxiety and depressive disorders meeting DSM-IV criteria, were receiving appropriate pharmacological treatment [8,9]. Indeed, psychological interventions are rarely delivered in this setting [9].

Different factors can be assumed to explain low rates of treatments, including patients’ opinions and preferences [10], under recognition of mental disorders [11], and, with regards to psychological interventions, limited availability of trained physicians [12,13], poor confidence by PCPs [9,14], and time constraints due to the setting [15,16]. It is possible that PCPs’ knowledge and attitude about treatments, also play an important role, but very few studies have investigated this issue. Aims of the present study are:

1) to assess PCPs’ opinions, knowledge and attitudes about the management of anxiety and depressive disorders;

2) to evaluate differences among PCPs subgroups and in particular comparing by gender, age, duration of clinical practice, and office location.

Assuming that a negative attitude or poor knowledge about treatments can diminish the chance to deliver it or to refer patients to the specialist, data from this research could allow the identification of specific educational needs and therefore the organization of tailored PCPs training on mental health issues.

## Methods

### Study design

The present cross-sectional survey was promoted by the Emilia Romagna Regional Programme “G. Leggieri” for the integration between Primary Care and Mental Health [17,18]. Emilia Romagna is a Northern Italian Region with, approximately, 4.405.500 inhabitants. It is sectored into 11 Local Health Units, with a Primary Care Department and a Mental Health Department each. To date, 3138 PCPs work in the Region. Four out of 11 Local Health Units of the Region (Bologna, Modena, Reggio Emilia, Rimini) were involved in the present study between October 2006 and December 2008. In order to avoid any kind of influence on PCPs practice, no specific education courses about depression and anxiety treatments were provided during the recruitment period.

PCPs were enrolled during psychiatric educational meetings organized by the Regional Programme “G. Leggieri”. This survey was authorized by the Primary Care Departments of the four Local Health Units involved and the steering committee of the Regional Programme “G. Leggieri”. These meetings were mandatory for PCPs as part of their continuous education programme. Each participant physician received the *Common Mental Disorders Questionnaire* and the *Depression Attitude Questionnaire*[19] with a cover letter explaining the aims of the study. Questionnaires were anonymous and participation in the survey was voluntary.

### Common mental disorders questionnaire (CMDQ)

“Common Mental Disorders” expression, indicates a not severe subgroup of anxious-depressive disorders, which frequently affected general population (almost 1 person on 5) and that are often managed in Primary Care. A team work including a psychologist (F.C.), a psychiatrist (M.M.) and a PCP (A.S.), developed an ad-hoc, self-reported questionnaire: the CMDQ aimed to investigate PCPs’ opinions and attitude toward patients with anxiety and depressive disorders.

The questionnaire is divided in 4 sections focus on the following areas:

1) *PCPs’ individual characteristics*: age, gender and professional details (previous specialization, years of clinical practice, numbers of attenders, group or solo practice, educational initiatives about Mental Health topics attended).

2) *Opinions, confidence and self-reported clinical behaviour in the management of patients*: personal confidence in dealing with anxiety and depressive disorders, considering relation with patients, diagnosis, pharmacological and psychological treatments. PCPs’ opinion about ADs and psychotherapy effectiveness, and the treatment they usually provided.

3) *Collaboration with Mental Health Services*: degree of satisfaction, opinions about obstacles and preference on type on collaboration with Mental Health professionals.

4) *Perceived needs for education and training on psychiatric issues*: estimation of PCPs’ own skills in managing anxiety and depressive disorders.

It was explained that “psychological interventions” is referred to psychotherapy, counselling and psychological support at general. Some others items instead were referred to “psychological interventions” at general, and others were specific for different interventions.

A 5-points Likert scale with score anchors of “strongly disagree/strongly agree” “not at all satisfied/completely satisfied”, measured each items; multiple choice questions were also presented.

### Depression attitude questionnaire (DAQ)

The DAQ [19] is a self-completion instrument comprising 20 statements about depression, concerning aetiology, course, treatment options, and role of the PCP, mental health specialist and nurse. Answers to each item are marked on a 100 mm visual analogue scale between “strongly disagree” (0 mm) and “strongly agree” (100 mm). The questionnaire has been used in several countries by various health professionals [20-22] like NHS direct nurses [23], practice nurses [24], district nurses [25], in-patient staff [26] and by psychiatrists [27].

An Italian version of DAQ with a three factor solution has been elaborated [28]. For the purpose of this study, only items 12, 13, 15, 16, 17, 18, 19 and 20 were considered (Additional file 1).

### Data analysis

PCPs individual characteristics and responses to closed-end items were analysed using general descriptive statistics, including numbers, proportions, means and standard deviations. For ease of presenting the 5-points Likert scales’ results were grouped together in three categories: “disagree”, “neutral”, “agree”. With regard to confidence the PCPs’ answers were grouped in: “very/extremely unconfident”, “neither confident nor unconfident” and “very/extremely confident”.

Between-group comparisons were made by gender, age, duration of clinical practice (more or less than 15 years), and the PCP’s office location (urban centre >100.000 inhabitants, urban centre <100.000 inhabitants, suburban, and rural/mountain areas). Comparisons were tested by the Student’s t test and one-way analysis of variance (least significant difference, LSD, Bonferroni, post hoc test), and p values less than or equal to 0.05 were considered statistically significant. For multiple post hoc comparisons assessing the influence of office location (4 subgroups), we adjusted the level of significance at 0.008 (0.05/6).

Statistical Package for the Social Sciences version 15.0 for Windows was used to carry out the analysis [29].

## Results

### Participants

**Figure 1:**
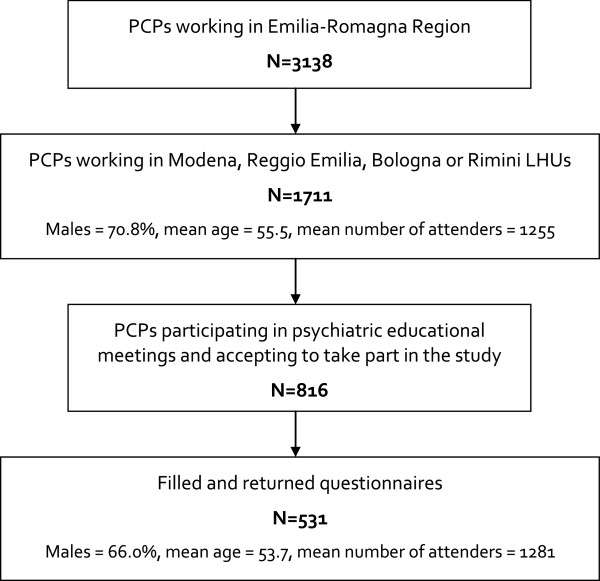
Flow diagram of the study.

Of the 816 questionnaires distributed, 531 were returned (response rate = 65.1%). The 66.0% of participants were males and had mean age of 53.7 ±5.1 years (ranging from 29 to 69). Mean duration of clinical practice in Primary Care was 22.0 ±8.2 years (range: 1 to 41). Forty point eight percent reported to work in a group practice. The mean number of attenders per PCP was 1281 ±358 (range 85 to 1970) (Figure 1). Seventy-three point four percent of PCPs attended at least one meeting or conference on psychiatric topics in the previous two years.

The most frequent physicians’ previous specializations were: Internal Medicine (9.2%), Geriatrics (7.5%), Gastroenterology (6.1%), Paediatrics (6.1%), General Medicine (5.8%), Pneumology (5.3%) and Cardiology (5.1%). Several other specializations were less frequent than 5.0%. Only 1.2% of PCPs had a specialization in Psychiatry and 2.2% in Psychology. Finally, 12.6% of PCPs reported to have no specialization.

### Opinions and confidence towards management

PCPs strongly agreed on ADs’ effectiveness in the treatment of depressive disorder, while opinions about their effectiveness for anxiety disorder and about psychotherapy for both conditions were less consistent (Table 1). Overall, only half of PCPs reported to feel confident in managing depressed patients and the rate of PCPs reporting “very/extremely” confidence was: 39.3% in recognition and diagnosis, 20.3% in pharmacotherapy, and 7.3% in delivering psychological interventions. With regards to anxiety disorder, these proportions increased to 67.2%, 55.1%, 33.9%, and 15.3%, respectively.

**Table 1:** PCP’s opinions about effectiveness of antidepressants and psychotherapy for treatment of anxiety and depressive disorders

**Statement**	**AD is effective for depressive disorder**	**Psychotherapy is effective for depressive disorder**	**AD is effective for anxiety disorder**	**Psychotherapy is effective for anxiety disorder**
Disagree	2.3	18.3	13.6	12.5
Neutral	12.5	33.5	34.8	26.1
**Agree**	**85.3**	**48.2**	**51.5**	**61.4**

From the DAQ answers analysis, 27.4% of PCPs thought it was rewarding to spend time looking after depressed patients (DAQ Item 15), and 92.9% strongly agreed with the statement “*working with depressed patients is heavy going*” (DAQ Item 13). Regarding the roles of PCPs and psychiatrists, 56.1% of PCPs strongly disagreed with the statement “*if depressed patients need antidepressants, they are better off with a psychiatrist than with a general practitioner*” (DAQ Item 17), and 68.0% of them stated that psychotherapy should be left to a specialist (DAQ Item 19). Only 20.0% of physicians considered psychotherapy not effective for depression (DAQ Item 16), and 37.0% of PCPs agreed with the statement “*if psychotherapies were more easily accessible, they would be more beneficial than drugs for most of the patients*” (DAQ Item 20).

### PCPs’ clinical behaviour

PCPs managed an average of 66.3% of patients suffering from anxiety and depressive disorders, while 32.6% are referred to a specialist. Pharmacotherapy was the intervention most frequently delivered by PCPs in the treatment of anxiety and depressive disorders (93.0%), followed by psycho-education (67.4%), generic psychological support (56.1%), and family intervention (33.2%). Structured psychological interventions such as counselling, behavioural intervention and problem solving were rarely provided (15.8%, 11.9% and 6.2% respectively).

### Comparisons by gender, age, years of practice, and office location

In comparison by gender, female often adopted psycho-education and environmental support, as well as pharmacological treatment. Compared to their male colleagues they felt less confident in treating depressive and anxiety disorders and perceived their knowledge in recognition, diagnosis and treatment of these disorders as less adequate (Table 2).

**Table 2:** PCPs’ socio-demographic characteristics and opinions: comparison by PCPs’ gender

	**Gender**		**Statistics**
	**Male**	**Female**	**(χ**^**2 **^**/F, df, p)**
**Age**, mean (sd)	55.0 (4.8)	51.2 (4.8)	59.902, 1, <0.001
**Medical practice**, years, mean (sd)	23.8 (7.9)	18.6 (7.9)	49.488, 1, <0.001
**Number of patients**, mean (sd)	1332.5 (325.7)	1184.7 (398.7)	19.873, 1, <0.001
**Intervention**			
Psychoeducation, %	63.8	74.2	4.213, 1, 0.040
Psychopharmacology, %	93.7	91.4	0.682, 1, 0.409
Behavioural, %	13.0	10.2	0.667, 1, 0.414
Problem solving, %	7.5	3.9	1.870, 1, 0.171
Counselling, %	16.2	15.6	0.021, 1, 0.884
Psychological support ,%	54.2	60.2	1.245, 1, 0.264
Family intervention, %	29.6	39.8	3.995, 1, 0.046
**Effectiveness of ADs**			
Depressive disorders, agree %	86.2	83.2	0.866, 2, 0.649
Anxiety disorders, agree %	50.7	52.5	0.163, 2, 0.922
**Effectiveness of psychotherapy**			
Depressive disorders, agree %	48.0	49.2	0.539, 2, 0.764
Anxiety disorders, agree %	56.4	71.5	11.45, 2, 0.003
**Confidence in the management**			
Depressive disorders, very/extremely confident %	53.7	39.5	7.133, 2, 0.028
Anxiety disorders, very/extremely confident %	71.6	58.9	6.366, 2, 0.041
**Adequate knowledge on CMD diagnoses**, agree %	47.9	36.5	4.683, 2, 0.096
**Adequate knowledge on CMD treatment**, agree %	43.0	29.6	6.987, 2, 0.030
**DAQ 15** M (SD)	49.0 (29.3)	42.4 (25.1)	6.446, 1, 0.011

List of abbreviations: *PCP *Primary Care Physician, *ADs* Antidepressant Drugs, *CMD *Common Mental Disorder, *DAQ *Depression AttitudeQuestionaire.

Note: Only *DAQ *items showing a statistical significance in the comparison were presented.

Comparisons by PCPs’ office location are showed in Table 3. Physicians working in rural/mountain areas, delivered more psychological support than those in suburb areas (χ^2^ = 8.245, df = 1, p = 0.004). Moreover, a higher percentage of PCPs working in rural/mountain area and urban centres with <100.000 inhabitants supported ADs effectiveness in the treatment of anxiety disorders compared to their colleagues working in urban areas (χ^2^ = 12.462, df = 2, p = 0.002 and χ^2^ = 13.737, df = 2, p = 0.001, respectively). Similar findings were found for ADs effectiveness in the treatment of depressive disorder without reaching the statistical significance.

**Table 3:** PCPs’ socio-demographic characteristics and opinions: comparison by PCPs’ office location

	**Urban centre > 100.000**	**Suburb area**	**Rural/Mountain area**	**Urban centre < 100.000**	**Statistics**
					**(χ**^**2 **^**/F, df, p)**
**Age**, mean (sd)	54.0 (5.0)	53.9 (4.3)	52.9 (6.0)	53.8 (5.3)	0.927, 3, 0.428
**Medical practice**, years, mean (sd)	22.3 (8.4)	22.2 (7.8)	20.8 (8.8)	22.4 (7.9)	0.889, 3, 0.447
**Number of patients**, mean (sd)	1252.0 (375.2)	1341.7 (306.1)	1295 (337.5)	1273.0 (386.7)	1.491, 3, 0.216
**Intervention**					
Psychoeducation, %	65.6	70.7	72.7	61.4	3.528, 3, 0.317
Psychopharmacology, %	93.3	90.2	91.9	96.0	2.694, 3, 0.441
Behavioural, %	14.4	16.3	10.2	7.9	3.991, 3, 0.262
Problem solving, %	10.0	6.5	6.1	3.0	3.998, 3, 0.262
Counselling, %	15.6	18.5	16.3	13.9	0.785, 3, 0.853
Psychological support, %	56.7	45.7	66.3	54.5	8.348, 3, 0.039
Family intervention, %	32.2	30.4	32.7	35.6	0.619, 3, 0.892
**Effectiveness of ADs**					
Depressive disorders, agree %	81.9	83.7	90.6	88.6	12.445, 6, 0.053
Anxiety disorders, agree %	44.0	49.5	59.4	58.1	21.207, 6, 0.002
**Effectiveness of psychotherapy**					
Depressive disorders, agree %	49.5	46.2	51.9	44.2	3.0318, 6, 0.805
Anxiety disorders, agree %	63.3	59.6	57.5	64.4	5.849, 6, 0.440
**Confidence in the management**					
Depressive disorders, very/extremely confident %	48.9	51.1	54.9	41.3	8.003, 6, 0.238
Anxiety disorders, very/extremely confident %	66.7	63.3	72.5	66.3	7.569, 6, 0.271
**Adequate knowledge on CMD diagnoses**, agree %	46.1	38.5	50.0	41.7	4.682, 6, 0.585
**Adequate knowledge on CMD treatment**, agree %	36.0	36.3	45.5	36.3	3.771, 6, 0.708
**DAQ 12** M (SD)	50.8 (27.7)	52.3 (28.9)	66.4 (23.9)	56.09 (28.1)	8.087, 3, <0.001
**DAQ 16** M (SD)	36.2 (27.8)	41.3 (26.5)	36.2 (26.9)	46.19 (29.5)	3.523, 3, 0.015
**DAQ 19** M (SD)	71.5 (27.5)	76.4 (26.6)	65.0 (27.9)	71.61 (26.8)	3.034, 3, 0.029

List of abbreviations: *PCP *Primary Care Physician, *ADs* Antidepressants Drugs, *CMD *Common Mental Disorder, *DAQ *Depression Attitude Questionnaire.

Note: Only *DAQ *items showing a statistical significance in the comparison were presented.

No differences were found in the comparison by age of PCPs and duration of clinical practice with regards to opinions, self-confidence, and clinical behaviour.

## Discussion

Despite the key role PCPs play in the management of anxiety and depressive disorders, only few information are available on their knowledge and attitude toward the treatment of these disorders. Aim of the present study was to assess the above issues in a large and representative sample of 531 PCPs working in a Northern Italy region. To our knowledge this investigation is the first allowing a direct comparison between PCPs’ answers on both anxiety and depressive disorders and thus it can evaluate differential educational needs for these two major diagnostic categories.

Regarding depressive disorders, the large majority of PCPs showed to rely on ADs’ effectiveness, while less than a half considered psychotherapy as effective. However, only about 1 out of 5 of the interviewed physicians, believed that psychotherapy is not effective for depression, indicating a general positive opinion toward psychological treatments. About 37.0% of PCPs thought that if psychotherapies were more easily accessible, it would be more beneficial than drugs for most patients. Our data are in line with previous studies reporting that many PCPs considered ADs as effective and sufficient in most cases and psychotherapy as a completion of treatment [15,20]. Andersson [30] stated that all PCPs interviewed agreed with the statement “*for patients with Major Depression, psychotherapy cannot replace drug treatment*”.

Regarding anxiety disorder a higher proportion of physicians relied on psychotherapy, while less agreement on ADs effectiveness was reported. PCPs might perceive anxiety disorder as less severe than depression, thus not needing pharmacotherapy.

In general, our data show that evidence based recommendations for the management of depressive and anxiety disorders [5,6] were not completely adopted by PCPs. As other studies conducted in Primary Care clearly proved, psychological interventions for Major Depression are highly effective, being superior to usual PCPs’ care and comparable to ADs [31,32]. Therefore, the underestimation of psychotherapy effectiveness become problematic in Primary Care because mild forms of depression are common and psychological interventions are essential for patients at low risk-benefit ratio with drugs such as old people, severe physically ill, pregnant women, etc.

Regarding confidence towards management, 49.0% and 67.2% of PCPs reported to be confident in the recognition and diagnosis of depressive and anxiety disorders, respectively. In contrast with Richards et al.’s [14] study, reporting a high proportion of PCPs feeling “most/very confident” in treating depression with drugs, our data show that only 20.3% of PCPs felt “very/extremely” confident in delivering pharmacotherapy for depression. The availability of well-tolerated drugs and their frequent use in Primary Care demonstrated by pharmaco epidemiological data [33], makes the results not easy to understand. Although the pharmacological treatment was the most delivered in our sample, only a minority of PCPs expressed full confidence about ADs. As expected and in line with literature [9,34], PCPs did not seem as keen on recurring to psychological treatments and a very low rate of physicians felt very confident with them. There are several factors that could determinate a scarce use of psychological interventions in Primary Care, such as limited time of consulting [35], PCPs’ inadequate training on non-pharmacological interventions [36,37] and PCPs’ attitudes and opinions on psychotherapy [15,16,20]. Indeed, in our study the majority of family practitioners agreed with the statement “*psychotherapy for depressed patients should be left to specialists*” (DAQ Item 19), suggesting that PCPs consider psychological interventions not completely part of their duty and that they should be carried out by specialists.

Our data show that family medicine education could benefit from specific tailored training on diagnosis and treatment of anxiety and depressive disorders in order to increase their perceived confidence in the management of these patients. In particular, it seems crucial to provide training on psychological interventions suited to Primary Care setting. In a previous study, it was demonstrated that a practical evidence-based counselling intervention could feasibly be taught during PCPs training and that trainees found it helpful in their future practices [38].

Considering comparisons, PCPs working in rural/mountain areas and female PCPs reported to adopt more frequently non-pharmacological treatments, as psycho-education, psychological support and family intervention, than urban areas and male PCPs. Indeed, a higher proportion of female doctors, compared to male colleagues, agreed that psychotherapy is an effective treatment for anxiety disorder. Our results are consistent with the few previous studies investigating both attitude and clinical behaviour according to office location and gender [14,39]. The more frequent use of psychological interventions reported by rural PCPs could be explained by the limited accessibility to mental health services in their area. It is possible that they decided to personally treat these patients including psychological aspects [40]. This might be consistent with the relatively higher confidence reported by rural PCPs on recognition, ADs and doctor-patient relationship, for both anxiety and depressive disorders, compared to colleagues in suburb and urban areas. Also, the prevalence of depression is higher in rural than in urban areas, probably because rural population tends to have characteristics that are strongly associated with depression, including poor health status, chronic disease, and poverty [41]. Therefore, rural PCPs are more likely to treat depressed patients, acquiring experience and becoming confident with this disorder.

With regards to gender differences, consistently with literature, our findings show that female physicians tend to spend more time with patients [42,43], pay more attention to relational aspects of care [44] and to be more likely to engage in counselling and conversations about social and family issues, compared to male physicians who seemed to pay more attention to technical aspects of care (physical examinations) [39,43]. A previous Italian study was in line with our results: female PCPs provide a higher number of follow-up visits for mild and stabilized cases which mainly required clinical monitoring [45]. Other studies in different settings show that women have higher odds of being less confident in identifying and managing medical conditions compared to men [40,46,47]. Similarly in our study, female PCPs reported to feel less confident in managing depressive and anxiety disorders and they perceived their knowledge about recognition and treatment of anxiety and depression less adequate than men. They also reported that evidence-based guidelines influenced their clinical practice [48]. So we suppose that guidelines’ higher influence on female PCPs, led them having a high critical perception of their knowledge on psychiatric issues. Particularly, they could be better aware about psychotherapy effectiveness for mild depressive and anxiety disorders and were not able to deliver “psychological intervention” after family medicine education.

### Limitations

This study has some limitations that may affect the generalizability of results. The socio-demographic and practice characteristics of the sample are comparable to regional PCPs population (personal communications by the four participating Primary Care departments). However, it is not possible to generalize these results to other countries with different healthcare organization or to whole Italian Primary Care: the Emilia Romagna Region is one of wealthiest area of Italy and has a well organized and developed health care system with a specific program to integrate Primary Care and Mental Health services [18]. Despite the high response rate, there might still be a non-response bias; the participation was asked during mandatory educational initiatives but PCPs can refuse to entry in the study. So physicians with poor interest in mental health issue and/or those with little or no confidence in management of mental disorders might have chosen not to participate, hence possibly overestimate our findings on comfort. However, our data showed low confidence rates and thus we think that this bias do not affect extensively our findings.

This study was based on a self-report investigation and did not evaluate PCPs clinical behaviour by objective measures. The relationship between opinions/attitudes and clinical behavior is controversial. Some studies suggested that attitude can affect the management of anxiety and depressive disorders, influencing clinical choice, use of psychological interventions and referral to psychologists [49], while other studies found that opinions are not good predictors of PCPs’ clinical practice [50].

We investigated ADs as a drug class and we have no information about the type of AD and prescribed dose. Considering that in Primary Care setting the average daily doses are usually lower than those recommended for the treatment of the major depression [33], our study does not evaluate the appropriateness of PCPs choice. Moreover, we have no available data on the use of benzodiazepines. Their prescription is very frequent in the Italian Primary Care setting [51,52] and it would be of interest to assess, at the same time, PCPs’ opinions and confidence about both ADs and benzodiazepines for the management of anxiety disorders. However, our study protocol did not allow this comparison and this issue requires further research.

There are very few published reliable instruments assessing PCP’s opinions, knowledge, and attitudes about mental disorders. An ad-hoc questionnaire was developed and subsequently tested in some pilot surveys to define the most useful items and a final version. We do not evaluate the reliability and the validity of the instrument.

## Conclusions

Our results suggest that an effort should be made to disseminate the most recent evidences on the management of depressive and anxiety disorders in Primary Care during and after the family medicine education. In particular, it should be emphasized the important role of psychological interventions for these disorders. It seems crucial to teach PCPs the basic skills for providing brief structured psychological support and to increase professional PCPs comfort in this area. Results concerning the poor confidence of PCPs in using ADs are unexpected and need further investigations. A qualitative research might be useful to light up on this topic and better address PCPs educational needs. Relevant differences in educational needs were found among PCPs subgroups, especially when analysing the sample by gender and office location. Training initiatives should take into account these differences and tailor adequate programs in order to work on specific point of weakness. For example, male PCPs should benefit to pay attention to psychological skills during continuing medical education.

## Abbreviations

ADs: Antidepressants; PCPs: Primary Care Physicians.

## Competing interest

The authors declare that they have no competing interests.

## Authors’ contributions

MMe, DB, AS and FC conceived and designed the study. FC, CS, PT and MMe analysed and interpreted the data. FC and MMe wrote the first draft of the manuscript. All authors contributed to the critical revision of the manuscript for important intellectual content. All authors read and approved the final manuscript.

## Pre-publication history

The pre-publication history for this paper can be accessed here:

http://www.biomedcentral.com/1471-2296/14/75/prepub

## Supplementary Material

Additional file 1 DAQ items. Click here for file
