# Supplementary material for: Primary care Physicians’ perspective on the management of anxiety and depressive disorders: a cross-sectional survey in Emilia Romagna Region
Source: BMC Fam Pract. 2013 Jun 7;14:75. doi: 10.1186/1471-2296-14-75 (PMC3688370; doi:10.1186/1471-2296-14-75)
Supplement: Additional file 1 — DAQ items. [file 1471-2296-14-75-S1.doc]

Appendix 1. DAQ items

| **DAQ’ items** |  |
| --- | --- |
| **1.** During the last 5 years, I have seen an increase in the number of patients with depressive symptoms.  *Strongly disagree______________________________________________________________________Strongly agree*  **2.** The majority of depression in general practice originates from patients' recent misfortunes.  *Strongly disagree______________________________________________________________________Strongly agree*  **3.** Most depressive disorders seen in general practice improve without medication.  *Strongly disagree______________________________________________________________________Strongly agree*  **4.** An underlying biochemical abnormality is at the basis of severe cases of depression.  *Strongly disagree______________________________________________________________________Strongly agree*  **5**. It is difficult to differentiate patients with unhappiness or a clinical depressed disorder that need treatment.  *Strongly disagree______________________________________________________________________ Strongly agree*  **6.** It is possible to distinguish two main group of depression: psychological / biochemical.  *Strongly disagree______________________________________________________________________ Strongly agree*  **7.** Becoming depressed is a way that people with poor stamina deal with difficulties.  *Strongly disagree______________________________________________________________________ Strongly agree*  **8.** Depressed patients are more likely to have experienced deprivation in early life than other people.  *Strongly disagree______________________________________________________________________Strongly agree*  **9.** I feel comfortable in dealing with depressed patients' needs.  *Strongly disagree______________________________________________________________________Strongly agree*  **10**. Depression reflects a characteristic response in patients, which is not amenable to change.  *Strongly disagree______________________________________________________________________ Strongly agree*  **11.** Becoming depressed is a natural part of being old.  *Strongly disagree______________________________________________________________________ Strongly agree*  **12.** The practice nurse could be a useful person to support depressed patients.  *Strongly disagree______________________________________________________________________Strongly agree*  **13.** Working with depressed patients is heavy going.  *Strongly disagree______________________________________________________________________Strongly agree*  **14**. There is little to be offered to those depressed patients who do not respond to what general practitioners do.  *Strongly disagree______________________________________________________________________ Strongly agree*  **15.** It is rewarding to spend time looking after depressed patients.  *Strongly disagree______________________________________________________________________Strongly agree*  **16.** Psychotherapy tends to be unsuccessful with depressed patients.  *Strongly disagree______________________________________________________________________Strongly agree*  **17**. If depressed patients need antidepressants, they are better off with a psychiatrist than with a general practitioner.  *Strongly disagree______________________________________________________________________ Strongly agree*  **18**. Antidepressants usually produce a satisfactory result in the treatment of depressed patients in general practitioner.  *Strongly disagree______________________________________________________________________ Strongly agree*  **19.** Psychotherapy for depressed patients should be left to a specialist.  *Strongly disagree______________________________________________________________________ Strongly agree*  **20.** If psychotherapy was freely available, this would be more beneficial than antidepressants, for most depressed patients.  *Strongly disagree______________________________________________________________________ Strongly agree* | |
